# The sex prevalence of lower limb varicose vein networks

**DOI:** 10.1016/j.jvsv.2024.101944

**Published:** 2024-07-06

**Authors:** Giulia Baldazzi, Mirko Tessari, Matilde Zamboni, Anselmo Pagani, Paolo Zamboni

**Affiliations:** a School of Vascular Surgery, University of Ferrara, Ferrara, Italy; b Department of Translational Medicine, University of Ferrara, Ferrara, Italy; c Vascular Diseases Center, University of Ferrara, Ferrara, Italy; d Vascular Surgery Unit, Hospital of Belluno, Belluno, Italy

**Keywords:** Varicose veins, Sex prevalence, Anterior accessory saphenous vein, Great saphenous vein, Venous valve

## Abstract

**Objective:**

To determine the sex prevalence of lower limb varicose networks fed by reflux of the great saphenous vein (GSV), anterior accessory saphenous vein (AASV), and small saphenous vein singularly or in combination.

**Methods:**

We scanned by the means of the same color Doppler ultrasound protocol 3000 lower limbs in 1500 consecutive patients, affected by symptomatic chronic venous insufficiency from 2013 to 2023. Limbs with normal venous function, incomplete scans, or that were affected by post-thrombotic syndrome, pelvic reflux, isolated perforator reflux, venous malformation, phlebolymphedema and Clinical, Etiological, Anatomical, Pathophysiological clinical class C5 and C6 were excluded from the final analysis.

**Results:**

Overall, 1072 patients—252 (23.5%) males and 820 (76.5%) females (*P* < .0001) matched for age (*P* = .692)—were included in the study for a total of 1956 limbs affected by primary chronic venous insufficiency, clinical class C2 to C4. The main finding was the significant prevalence of varicose networks fed by reflux of the AASV alone (odds ratio [OR], 1.96; 95% confidence interval [CI], 1.26-3.06; *P* = .001) or combined with GSV (OR, 1.84; 95% CI, 1.34-2.52; *P* = .0002) in females. In contrast, GSV insufficiency alone was significantly prevalent in males (OR, 0.54; 95% CI, 0.43-0.68; *P* < .0001). No significant sex differences regarding SSV reflux were detected. Moreover, we considered the presence of competent terminal valve (TV+) at the level of the saphenofemoral junction, which resulted more significantly present in female (OR, 1.57; 95% CI, 1.12-2.19; *P* = .0083); to the contrary incompetent terminal valve (TV−) was more common in males (OR, 0.64; 95% CI, 0.46-0.89; *P* = .0083). Finally, considering reflux in the AASV territory in the presence of a TV+, a strong prevalence in females was detected (OR, 2.28; 95% CI, 1.48-3.52; *P* = .0002), whereas males developed reflux along the GSV when a concomitant TV− was present (OR, 0.62; 95% CI, 0.41-0.94; *P* = .0244).

**Conclusions:**

The analysis of the lower limb varicose networks highlights that reflux along the AASV alone, in presence of a TV+ at the junction or coupled with GSV insufficiency, is more prevalent in females. In contrast, GSV resulted the main trunk feeding varicose veins in males, in particular when a TV− was detected. Our findings suggest that females could be more prone to developing varicose veins with an ascending mechanism, whereas in males the descending one seems to be more common.

**Article Highlights**

• **Type of Research:** Single-center, prospective study
• **Key Findings:** Reflux in the anterior accessory saphenous vein territory, alone or combined with great saphenous vein incompetence, is almost two-fold more prevalent in females. To the contrary, great saphenous vein reflux resulted more prevalent in males. Females are more likely to develop varices in presence of a competent junction (*P* = .0083).
• **Take Home Message:** Reflux along the anterior accessory saphenous vein, in presence of a competent terminal valve, is significantly more prevalent in females (*P* = .0002).

The influence of gender (social constructs) and sex (biological constructs) in medicine has grown in importance in the last decades. Traditionally, the origin of the gender-specific medicine, defined as the study of how diseases differ between men and women in terms of prevention, clinical signs, therapeutic approach, prognosis, and psychological and social impact, dates back to last century.^1^^,^^2^ Since then, the commonest global causes of death and morbidity have been characterized considering both sex and gender as importance modifier factors; in a review published in *The Lancet*, many pathologies such as heart disease, cancers, stroke, and diabetes were compared in men and women.^3^

Worldwide, chronic venous insufficiency (CVI) represents the most frequent vascular disease with an estimated prevalence ranging between 15% and 80%^4^; furthermore, the incidence varies between countries, as highlighted by the Edinburgh Vein Study^5^ and the 24-cities Cohort Study in Italy.^6^ Considering the C class of the Clinical, Etiological, Anatomical, Pathophysiological classification,^7^ C2 (varicose veins), C3 (edema), and C4 (changes in skin and subcutaneous tissue) prevalence is approximately 19%, 8%, and 4%, respectively,^8^ with the great saphenous vein (GSV) and the small saphenous vein (SSV) as the most affected segments.

Multiple systemic factors, both anatomical and dynamic, were associated with CVI^9^; the sex prevalence of female for the development of varicose veins and primary CVI is well-established.^5^^,^^10^ However, varicose networks still represent a complex argument: the influence of singular factors, as sex, on varicosity development in superficial vein territories (GSV, SSV) has never been investigated. Moreover, the relevant role of the anterior accessory saphenous vein (AASV), as a truncal vein of the superficial compartment and not just a GSV tributary, has been recognized, quite recently^11^; as a matter of fact, Meissner et al^12^ restored the importance of the AASV, recommend abandoning the term accessory and using the more appropriate name anterior saphenous vein (ASV). The aim of this study was to determine the sex prevalence of varicose networks among GSV, SSV, and ASV territories and their combination.

## Methods

We performed a prospective analysis on 3000 consecutive patients affected by symptomatic CVI (1500 limbs) referred to the Vascular Lab of the Vascular Disease Centre at the University of Ferrara (Italy) between 2013 and 2023. Exclusion criteria were normal limbs, incomplete investigation, post-thrombotic syndrome, pelvic reflux, isolated perforator reflux, venous malformation, phlebolymphedema and CEAP clinical class C5-C6. We choice to exclude C5-C6 patients, as a relevant proportion was less compliant in undergoing the color Doppler ultrasound protocol in standing position and, for this reason, many ultrasound (US) examinations were not enough detailed to be included. C1 patients, instead, were not considered in this study, because they are usually not referred to our surgical vascular laboratory.

By adopting a protocol of color Doppler US investigation previously reported,^13^, ^14^, ^15^ we scanned both lower extremities in patients in standing position (LOGIQ S8 with XDclear, GE HealthCare Technologies Inc., Chicago, IL) with a linear probe (frequency range, 7-12 MHz).

Informed, written consent and agreement to publication of images was obtained by every patient included in the study. The study was approved by the local ethical committee.

### Color Doppler US protocol

#### US anatomical landmarks

**Fig 1:**
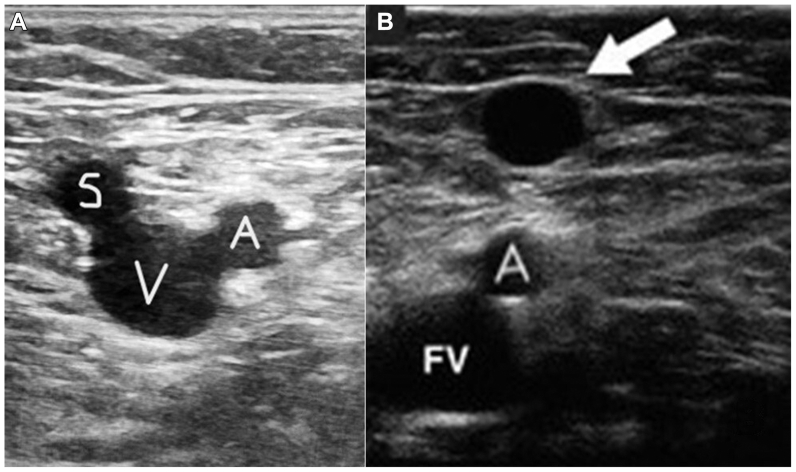
**(A)** Identification of the saphenofemoral junction (SFJ) using the Mickey Mouse sign during ultrasound (US) scan. *A*, common femoral artery; *S*, SFJ; *V*, femoral vein. **(B)** Identification of the anterior saphenous vein (ASV; arrow) thanks to its projection on the deep vessels at the US scan (alignment sign). *A*, *s*uperficial femoral artery; *FV*, femoral vein.

The protocol begins at the saphenofemoral junction (SFJ). The patient was invited to spend some time standing on an elevated platform, a comfortable position for the investigator. In order to identify the SFJ, the transverse US scan allows to recognize the three main vessels of the groin using the Mickey Mouse sign (Fig 1, *A*).

The GSV is usually the easiest vein to identify, because it pierces the fascia and connects through the SFJ with the femoral vein. At that level, constantly the terminal valve (TV) can be visualized. Downward, the GSV passes the E point,^16^ over the adductor longus muscle at 3 to 5 cm from the junction, and runs medially in the limb, between the fasciae, where it can be easily recognized by the typical image of the Egyptian eye.^17^, ^18^, ^19^ Instead, the Egyptian eye of the ASV runs from the mid-thigh to the groin. On US examination, it could be identified easily by its position (lateral to the GSV) and its projection over the deep vessels (alignment sign)^19^ (Fig 1, *B*).

Last but not least, the examination moves to the popliteal region, for the investigation of the SSV, the interfascial vein of the leg that runs at the back of the calf and can again be recognized by the Egyptian Eye. We investigated the junction between the SSV and the gastrocnemius and/or popliteal vein, also in cases where the junction was located above the popliteal fossa. Finally, according to a consensus conference, we examined all the veins running above the superficial fascia, and we considered and analyzed the saphenous tributaries.^19^

#### Hemodynamic assessment at the junctions

**Fig 2:**
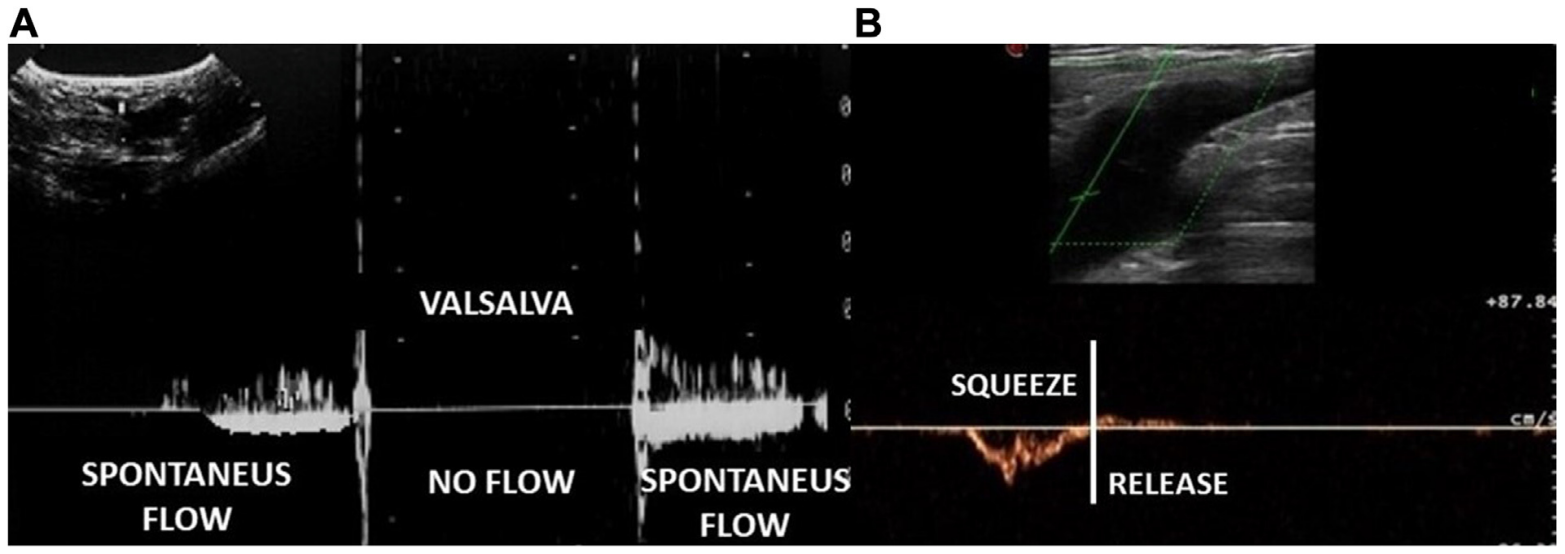
Physiological flow pattern at the saphenofemoral junction (SFJ). **(A)** Valsalva's maneuver effect on venous flow with the probe placed on the femoral side of the terminal valve (TV). From left to right: spontaneous venous flow before application of Valsalva's maneuver; arrest of the flow during the maneuver due to the closure of the competent TV that avoids reflux into the great saphenous vein (GSV) when pressure in the abdomen cavity increases; release of Valsalva with reinstatement of the spontaneous flow. **(B)** Physiological flow pattern with the probe placed on the femoral side of the TV during squeezing of the calf. From left to right: when the calf is squeezed (muscular systole), we appreciate a flow through the SFJ to the femoral vein; at the release of the calf (muscular diastole), no flow is detected (TV closure).

**Fig 3:**
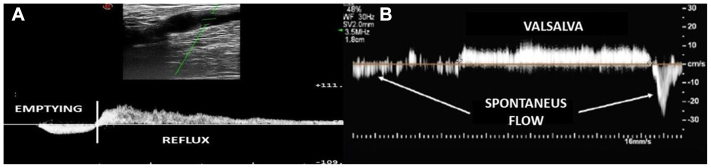
Pathological flow pattern with reflux in the great saphenous vein (GSV). **(A)** The probe is positioned on the femoral side of the terminal valve (TV). When the calf is squeezed (muscular systole), we appreciate the emptying of the saphenofemoral junction (SFJ) into the femoral vein; at the release of the calf (muscular diastole), a reflux was detected (TV incompetence). **(B)** The probe is positioned on the femoral side of the TV. During the Valsalva's maneuver, we detect a reflux at the level of the SFJ due to the inability of the TV to properly close (TV incompetence).

With the US sample placed at the femoral side of SFJ, the protocol starts assessing the competence of the GSV TV, by using two different maneuvers: the Valsalva (Fig 2, *A*) and calf squeezing (Fig 2, *B*), respectively. When at least one of the two maneuvers described resulted negative for reflux, we considered the TV as competent (TV+)^14^^,^^15^ (Fig 2). In contrast, when both maneuvers demonstrated reflux of >0.5 s, as described by Labropoulos et al^20^ and underlined by the clinical practice guideline of multiple vascular societies,^21^^,^^22^ we considered the TV incompetent (TV−), as shown in Fig 3.^14^^,^^15^ As far as the saphenopopliteal junction is concerned, we put the Doppler sample on the popliteal/gastrocnemius side of the junction; the presence of reflux was elicited either by squeezing or the so-called Paranà maneuver.^12^

#### Investigation of the varicose networks

**Fig 4:**
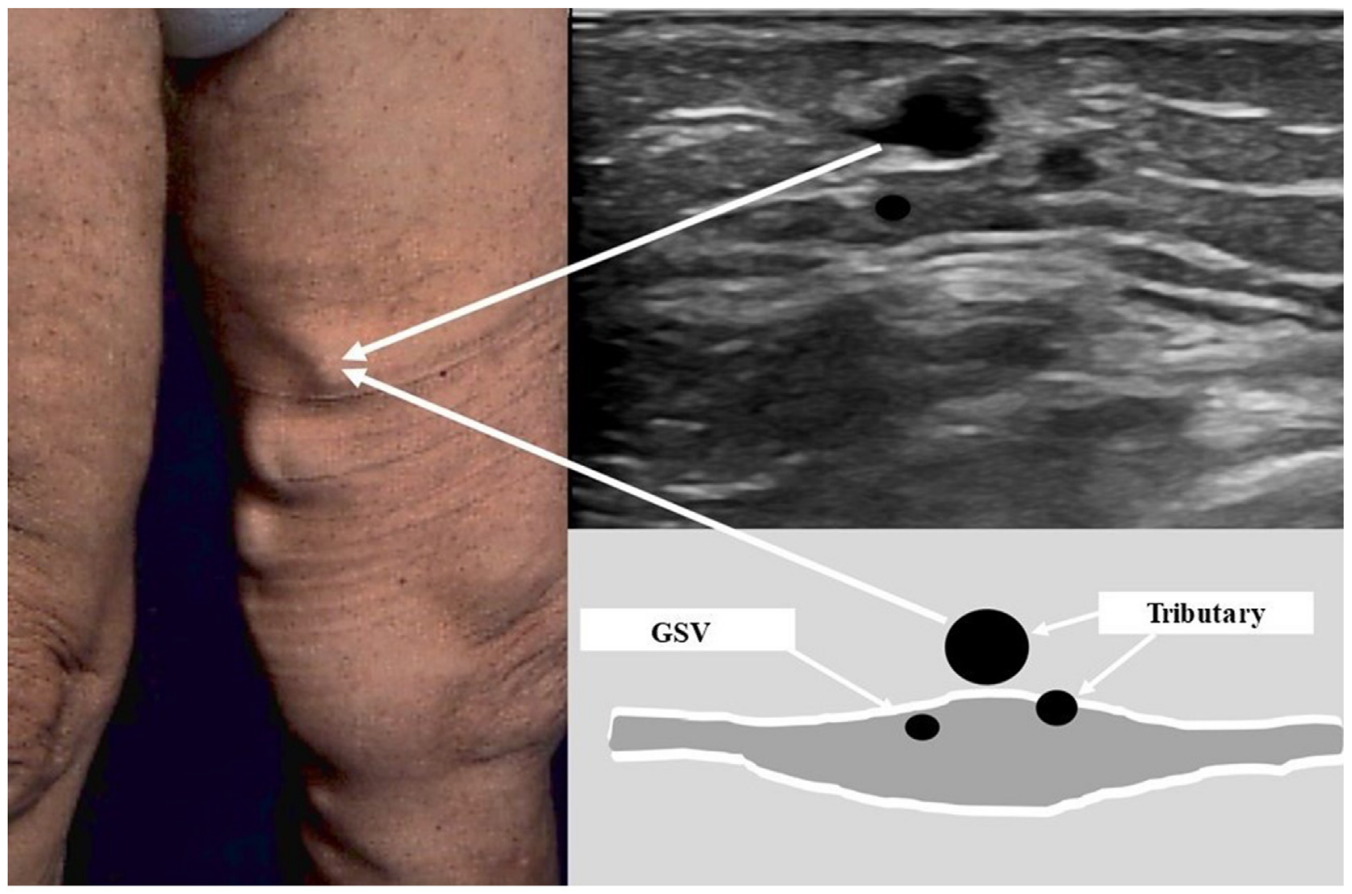
Example of identification of varicose tributaries of the great saphenous vein (*GSV*).

Once the GSV, ASV, and SSV were identified and assessed for reflux, we continued to scan them along the lower extremity in the transversal access, following the Egyptian eye imaging biomarker. Each of their tributaries were investigated for reflux at their origin, locating the tributary at one of the three saphenous trunks (Fig 4). To recognize a physiological or pathological flow, pulse wave Doppler mode was activated and each of the tributaries were tested. Venous flow was elicited by the calf squeezing maneuver.^19^ A tributary was considered incompetent when the reflux flow was >0.5 s.^20^

### Statistical analysis

All records regarding patient demographics and anatomical and hemodynamic data characterizing saphenous reflux and varicose networks were collected, tabulated and subdivided in male and female patients' cohort in a Microsoft Excel database (Microsoft Corporation, Redmond, WA). All categorical variables are reported as absolute frequency and relative frequency (%); continuous variables are reported as mean and standard deviation. Categorical variables were tested applying the χ^2^ test. We considered a *P* value of <.05 to be statistically significant. For the analysis, the software Jamovi (version 2.4) was used.^23^

## Results

After applying the above exclusion criteria, 1072 patients affected by primary CVI were included in the study and subdivided according to sex. The well-known prevalence of varicose veins in females was confirmed: 820 (76.5%) females vs 252 males (23.5%) (*P* < .0001). The two groups were homogenous for age (mean age, 57±13.5 years old [females] and 56±13.9 years old [males]; *P* = .692). Overall, 1956 limbs were considered, in particular 1524 limbs (77.91%) in females and 432 (22.09%) in males. Further analysis of the varicose networks attributed to the three main saphenous trunks involved by reflux are given in Table I, according to sex and the number of limbs.

**Table I:** Varicose networks prevalence in both groups subdivided according to sex and considering total limbs affected by chronic venous insufficiency (CVI) for the analysis

Varicose networks	Total limbs (n = 1956)				
	Male (n = 432)	Female (n = 1524)	OR	CI	*P* value
ASV	25 (5.8)	167 (11)	1.96	1.26-3.06	**.001**
ASV + GSV	52 (12)	306 (20.1)	1.84	1.34-2.52	**.0002**
ASV + SSV	3 (0.7)	9 (0.6)	0.85	0.23-3.15	.8074
GSV	300 (69.4)	841 (55.2)	0.54	0.43-0.68	**<.0001**
GSV + SSV	18 (4.2)	62 (4.1)	0.98	0.57-1.67	.9274
SSV	34 (7.9)	139 (9.1)	1.17	0.79-1.73	.4196

*ASV,* Anterior saphenous vein; *CI,* confidence interval; *GSV,* great saphenous vein; *OR,* odds ratio; *SSV,* small saphenous vein.

Values are number (%) unless otherwise noted.

Boldface entries indicate statistical significance.

The main finding was the significant prevalence, in the female group, of varicose networks fed exclusively by reflux of the ASV (odds ratio [OR], 1.96; 95% confidence interval [CI], 1.26-3.06; *P* = .001) or by contemporary insufficiency of the ASV and GSV (OR, 1.84; 95% CI, 1.34-2.52; *P* = .0002). In contrast, GSV reflux alone was significantly prevalent in males (OR, 0.54; 95% CI, 0.43-0.68, *P* < .0001). No significant differences between the two groups have been recorded when SSV was affected by reflux due to an incompetent SPJ (34 and 139 cases in males and females, respectively; *P* = .4196). Moreover, we highlighted the presence of a TV+ or a TV− at the SFJ. We performed a subgroups analysis, considering reflux in the GSV or ASV, alone or combined, in both the male and female groups (Table II).

**Table II:** Prevalence of competent terminal valve (TV+) or incompetent terminal valve (TV−) in both groups, according to sex division, and subanalysis considering reflux in the main saphenous trunks (anterior saphenous vein [ASV] and great saphenous vein [*GSV*]) alone or combined

Varicose networks	Total limbs (n = 1956)				
	Male	Female	OR	CI	*P* value
Overall	195 (9.97)	691 (35.33)	1.01	0.81-1.25	.9406
TV+	121 (62.05)	497 (71.92)	1.57	1.12-2.19	**.0083**
TV−	74 (37.94)	194 (28.07)	0.64	0.46-0.89	**.0083**
ASV reflux					
TV+	28 (14.35)	191 (27.64)	2.28	1.48-3.52	**.0002**
TV−	20 (10.25)	47 (6.8)	0.64	0.37-1.11	.1095
GSV reflux					
TV+	76 (38.97)	220 (31.38)	0.73	0.53-1.02	.0626
TV−	39 (20)	93 (13.45)	0.62	0.41-0.94	**.0244**
ASV + GSV reflux (lambda presentation)					
TV+	17 (8.71)	86 (12.44)	1.49	0.86-2.57	.1537
TV−	15 (7.69)	54 (7.81)	1.02	0.56-1.85	.9551

*ASV,* Anterior saphenous vein; *CI,* confidence interval; *OR,* odds ratio; *SSV,* small saphenous vein.

Values are number (%) unless otherwise noted.

Boldface entries indicate statistical significance.

Even if TV competence was not reported in all patient records, we included in the analysis an overall of 886 TV (195 (9.97%) males and 691 (35.33%) females) of the total of 1956 limbs. Considering the dichotomy TV+/ TV−, our data clearly showed a significant prevalence of TV+ in the female cohort as compared with males (OR, 1.57, IC 1.12-2.19; *P* = .0083). Moreover, in females, when the ASV represented the unique insufficient trunk, the presence of a TV+ was more common (OR, 2.28; 95% CI, 1.48-3.52; *P* = .0002); whereas in males, TV− was significantly associated with GSV insufficiency (OR, 0.62; 95% CI, 0.41-0.94; *P* = .0244).

## Discussion

Although the greater prevalence of varicose veins in females is well-known, their distribution in the GSV, ASV, and SSV, according to sex, has been not investigated to date. The main finding of our study is the significative prevalence of varicose veins fed by reflux of the ASV in females, either when the GSV is normal or when both segments are insufficient. These results are very interesting because the patient population is comparable in age, and they could indicate a predisposition of the female sex to develop varicose networks fed by reflux of the ASV itself. Unfortunately, in the general healthy population, it has never been described if the anatomical entity of the ASV is more prevalent in females. Concerning the GSV, its prevalence results significantly more pronounced in males.

Our findings have important clinical implications because one of the major factors causing recurrencies after endothermal ablation of the GSV is represented by reflux from the ASV. For instance, in a patient population largely composed by women (approximately 94%), Baccellieri et al^24^ reported that concomitant incompetence of the ASV or its direct confluence into the SFJ (OR, 1.5) could represent an indication to simultaneous radiofrequency ablation of both vessels, owing to the greater risk of recurrent varicose veins at 1 year after the procedure. From this point of view, cases with varicose networks fed by the combination of reflux of both GSV and ASV could represent a challenging treatment, also according to our findings. The prevalence of patients with both GSV and ASV insufficiency was 20% in female's and 12% in male's cohort. However, to our knowledge, no studies regarding differences in the recurrence rate based on the sex distribution of saphenous trunks reflux are present in literature. Finally, regarding varicose veins fed by reflux of the SSV, we did not find any significant differences between the two groups.

One more important finding of our study is that there is a significant prevalence of a TV+ in the female group, even when an ASV reflux is detected; in contrast, in the male group, the presence of TV− significantly determines reflux in the GSV. Our data, in fact, suggest a possible ascending origin of the varicose veins in females: this theory states that the varicose disease process could develop in the lower part of the leg and propagate cranially at the level of the main saphenous trunks, without involving the junction.^25^ Moreover, based on this theory, some authors^26^ supported the Ambulatory Selective Variceal Ablation under Local Anesthesia as a minimally invasive treatment for early stages of CVI that aims to remove varicose tributaries, considered to be at the origin of incompetence, without treating the saphenous trunk.^27^ However, in this systematic review, Richards et al^26^ did not perform a sex-oriented subanalysis and no other data supporting the ascending theory, according to sex, are present in literature so far.

Our study cannot explain why females are more likely to develop varicose veins despite a TV+. Speculatively, we need more data about gender peculiarities, in the hypothesis that something related to pregnancies, hormonal asset, oral contraceptives etc. may act on the vein wall and favors dilation of the networks above the superficial fascia^28^; thus, the insufficient tributaries could favorite a retrograde flow along the saphenous trunks. Furthermore, according to our data, the traditional vision of the descending theory, which supports that CVI could be secondary to a proximal disease (SFJ incompetence), seems more tailored to the male sex, where an incompetent SFJ is present in almost 40% of our patients.^25^

Some could argue that patients with TV− should have an increased risk of saphenofemoral reflux. In a recent review, it has been found that male sex and use of anticoagulation could be predictors of long-term failure of endovascular thermal ablation.^29^ Speculatively, this finding could be related to the increased presence of TV− we found in males.

The main limitation of our study is the lack of a control group, not affected by CVI, to understand if ASV is a segment anatomically more prevalent in the females. A further epidemiological limitation of our study is that the patient cohort is largely composed of females, owing to the high number of requests for consultation; this finding could be a consequence of the cosmetic and social problem of the varicose veins particularly perceived by females, as compared with males.

## Conclusions

Analysis of the lower limb varicose networks highlights that reflux along the ASV alone, in the presence of a TV+ at the junction or coupled with GSV insufficiency, is more prevalent in females. Our findings might support the recent idea that the ASV cannot be more considered an accessory segment but a well-defined truncal vein entity of the interfascial compartment. In contrast, GSV results the main trunk feeding varicose networks in males, particularly in the presence of TV+. Our findings suggest that females could be more prone to develop varicose veins with an ascending mechanism, whereas in males the descending mechanism seems more common. Surprisingly, the routes of reflux causing varicose veins are significantly different between the two sexes with a possible and intriguing ascending modality of the disease progression in the female group, never reported so far. However, further studies investigating also gender-specific factors and anatomy of ASV in people unaffected by CVI are warranted.

## Author Contributions

Conception and design: GB, PZ

Analysis and interpretation: GB, MZ, PZ

Data collection: GB, MT, MZ, AP

Writing the article: GB, MZ, AP, PZ

Critical revision of the article: MT, MZ, PZ

Final approval of the article: GB, MT, MZ, AP, PZ

Statistical analysis: GB

Obtained funding: PZ

Overall responsibility: PZ

## Disclosures

None.
